# Expression and immunolocalisation of *TpFABP* as a candidate antigen for the serodiagnosis of rabbit *Taenia pisiformis* cysticercosis

**DOI:** 10.1051/parasite/2013053

**Published:** 2013-12-12

**Authors:** Deying Yang, Lin Chen, Yue Xie, Xuhang Wu, Xiang Nong, Xi Peng, Weimin Lai, Xiaobin Gu, Shuxian Wang, Xuerong Peng, Guangyou Yang

**Affiliations:** 1 Department of Parasitology, College of Veterinary Medicine, Sichuan Agricultural University Ya’an 625014 China; 2 College of Veterinary Medicine, Sichuan Agricultural University Ya’an 625014 China; 3 College of Life and Basic Science, Sichuan Agricultural University Ya’an 625014 China

**Keywords:** *Taenia pisiformis*, Rabbit, Fatty acid-binding protein, Immunolocalisation, Immunodiagnosis

## Abstract

The larval stage of *Taenia pisiformis*, also known as *Cysticercus pisiformis*, is the causative agent of cysticercosis and the cause of severe health problems in rabbits that negatively impacts on husbandry production. To date, there is no fast detection method to identify early infections in rabbits. In the present study, a new dot-ELISA-based on an endogenous antigen fatty acid-binding protein (*FABP*) was developed for the detection of cysticercosis, and its potential was then evaluated using test serum samples. Immunolocalisation showed that *T. pisiformis FABP* (*TpFABP*) localised to the parenchyma of the bladder wall of the cysticercus and perinuclear cytoplasm of parenchyma of the adult parasite. After cloning and expression, recombinant *TpFABP* (r*TpFABP*) protein was used for serodiagnosis of *T. pisiformis* infection in rabbits by dot-ELISA. The antibody was detected 14 days post-infection in rabbits experimentally infected with *T. pisiformis*. Based on the necropsy results, the sensitivity and specificity of 169 serum samples tested by r*TpFABP* dot-ELISA were found to be 98.2% (54/55) and 92.1% (105/114), respectively. These data suggest that the dot-ELISA developed in this study has potential for detection of *T. pisiformis* infection in rabbits.

## Abstract (fr)

Le stade larvaire de *Taenia pisiformis*, également connu sous le nom *Cysticercus pisiformis*, est l’agent causal de la cysticercose et la cause de graves problèmes de santé chez les lapins, ayant un impact négatif sur la production d’élevage. À ce jour, il n’existe aucune méthode de détection rapide pour identifier les infections précoces chez les lapins. Dans cette étude, un nouveau dot-ELISA basé sur une protéine de liaison aux acides gras antigéniques endogènes (*FABP*) a été développé pour la détection de la cysticercose, et son potentiel a ensuite été évalué en utilisant des échantillons de sérum. L’immunolocalisation a montré que la protéine *FABP* de *T. pisiformis* (*TpFABP*) se localisait au niveau du parenchyme de la paroi du cysticerque et du cytoplasme périnucléaire du parenchyme du parasite adulte. Après clonage et expression, la protéine recombinante *TpFABP* (*rTpFABP*) a été utilisée chez les lapins pour le diagnostic sérologique par dot-ELISA de l’infection à *T. pisiformis*. L’anticorps a été détecté 14 jours après l’infection chez des lapins infectés expérimentalement avec *T. pisiformis*. Sur la base des résultats d’autopsie, la sensibilité et la spécificité de 169 échantillons de sérum testés par *rTpFABP* dot-ELISA ont été respectivement de 98 % (54/55) et 92 % (105/114). Ces données suggèrent que le dot-ELISA développé dans cette étude a un potentiel pour la détection de l’infection à *T. pisiformis* chez les lapins.

## Introduction

Cysticercosis, an infection caused by the larvae of *Taenia pisiformis* (Bloch, 1780) [27], is one of the most common parasitic disease in rabbits [11]. During the life cycle of *T. pisiformis*, the cysticerci present in the abdominal cavity of infected rabbit are ingested by a definitive host (canids and felines), following which the adult *T. pisiformis* individuals parasitises and matures in the host small intestine [2, 23]. The gravid proglottids of *T. pisiformis* released from infected dogs are in turn ingested by rabbit through contaminated food or water. The proglottids discharge oncospheres in the rabbit intestine and penetrate the intestinal mucosa and blood vessels. The oncospheres reach the liver parenchyma, then migrate to liver capsule, greater omentum and mesentery and develop into cysticerci [18, 20]. China is the world’s largest producer of rabbits [5], and *T. pisiformis* severely affects rabbit breeding. Rabbits infected with *T. pisiformis* are emaciated and have weak resistance to other diseases; in particular, it can also cause death especially for breeding rabbit.

The rapid and accurate detection of cysticercosis in rabbits is crucial for arresting its negative impact on husbandry production. In general, as there are no obvious early clinical symptoms in rabbits infected with *T. pisiformis*, it is a major challenge to control this disease. The presence of *T. pisiformis*-specific antibodies in serum from infected rabbits can provide the foundation for detection of this parasite [6, 29]. Crude antigens from oncospheres or mature metacestodes have been used in previous studies [6, 29]. However, due to the limited availability of crude parasite antigens, only a few serologic tests have been used to detect anti-*T. pisiformis* antibodies, including enzyme-linked immunosorbent assay (ELISA) and indirect fluorescent antibody test (IFAT) [6, 29]. In addition, the standard ELISA and IFAT methods are too complex to be used routinely under field conditions. Keeping these considerations in mind, dot-ELISA is one of the better serodiagnostic strategies due to its sensitivity and convenience.

Fatty acid-binding proteins (*FABP*s), multigenic cytosolic proteins are found in most animal groups. They are involved in the uptake and transport of hydrophobic ligands to different cellular fates [10, 13]. In helminthic parasites, *FABP*s are proven to be involved in acquisition and utilisation of host-derived hydrophobic substances, as well as in signalling and cellular interactions [16]. In the present study, a new *FABP* homologue *TpFABP*, from *T. pisiformis* was cloned and expressed and its immunolocalisation was then analyzed. Based on these results, a new recombinant *FABP* (r*TpFABP*) protein-based dot-ELISA was developed for the serodiagnosis of *T. pisiformis* infections in the rabbit industry.

## Materials and methods

### Ethics statement

All animals were handled in strict accordance with animal protection law of the People’s Republic of China (a draft of an animal protection law in China released on September 18, 2009) and the National Standards for Laboratory Animals in China (Laboratory animal – Standards and monitoring for parasitology, GB 14922.1-2001, executed on May 1, 2002). All experiment protocols were conducted according to the principles set forth in the Guide for the Care and Use of Laboratory Animals, Veterinary College, Sichuan Agricultural University, China.

### *TpFABP* amplification and structural prediction

Total RNA was isolated from mature metacestodes (provided by the parasitology laboratory at the Sichuan Agricultural University, China) using Trizol reagent (Invitrogen, Shanghai, China) according to the manufacturer’s instructions. The cDNA was obtained using the SuperScript Double-Stranded cDNA Synthesis kit (Invitrogen, Shanghai, China) following the manufacturer’s protocol. Based on the cDNA sequence of *T. solium FABP* (GenBank: DQ273765), the gene-specific primers for *TpFABP* were designed as follows (letters in parentheses represent the code of degenerate primers): F1 5′-ATGGAGSCATTCMTY(C)GKW(T)ACCTGGA-3′, R1 5′-TCCCTTACRY(T)CMCY(C)Y(T)TW(T)RMGTAGKTTC-3′. PCR was performed in a 25 μL final volume, including 12.5 μL of PCR mixture (Invitrogen, Shanghai, China), 0.4 μM of each primer (forward and reverse), 1 μL of cDNA template and 9.5 μL ddH_2_O. The amplification conditions consisted of an initial denaturing step at 94 °C for 5 min, followed by 35 cycles of amplification, 94 °C for 50 s, 56 °C for 45 s, and 72 °C for 50 s and a final extension step at 72 °C for 10 min. The PCR products were cloned into pMD19-T vector (TaKaRa, Dalian, China), and sequenced using an ABI PRISM^TM^ 377XL DNA Sequencer (ABI, Foster City, USA). The new *TpFABP* sequence was deposited in GenBank with Accession Number GU205472.

BepiPred 1.0 server (http://www.cbsdtu.dk/services/BepiPred/) was used to predict the location of linear B-cell epitopes [17]. PredictProtein (http://www.predictprotein.org/) was used to infer the secondary structures [22]. The alignment of *TpFABP* amino acid sequences with those of other Taeniidae cestodes and *Oryctolagus cuniculus* was performed using ClustalX 1.83 software [25], and the MegAlign program of DNAstar software package [4] was utilised to calculate the percentage identities.

### r*TpFABP* expression and western blotting

The expression sequence of *TpFABP* was amplified by F2 5′-GGGATCCATGGAGGCATTCCTCGGTA-3′ and R2 5′-CGCTCGAGTTACGTCCCTTTAAAGTAGGTTC-3′ using the same PCR conditions described above. The PCR products were subcloned into the *Bam*H1 and *Xho*l sites of the expression vector pET32a (Novagen, Darmstadt, Germany) and expressed in *Escherichia coli* BL21 (DE3) induced by 0.6 M isopropyl-*β*-d-thiogalactoside (IPTG). The *TpFABP* fusion proteins (fused with the Trx-Tag™ thioredoxin) were dissolved using 8 M urea, purified on an Ni-IDA sefinoseTM resin (Bio-Rad, California, USA), and the concentration of the purified protein was determined by a Biophotometer (Eppendorf, Hamburg, Germany) using a BCA Protein Assay Kit (Beyotime, Haimen, China) according to the manufacturer’s instructions.

The r*TpFABP* protein was separated on a 12% sodium dodecyl sulphate polyacrylamide gel electrophoresis (SDS-PAGE), and transferred to a nitrocellulose (NC) filtre membrane (Sigma, San Francisco, USA) by electroblotting. The membrane was washed three times for 5 min with 0.01 M phosphate buffer solution (PBS), and blocked with 5% non-fat milk powder in 0.01 M PBS for 2 h at room temperature. The rabbit antisera was probed with 1:100 dilution, and added directly to the blocking solution (including 5% non-fat milk powder and 0.01 M PBS) at 4 °C overnight. The rabbit *T. pisiformis* antisera were sourced from animals at 50 days post-experimental infection (provided by the laboratory of parasitology in Sichuan Agricultural University). The membrane was then washed three times with PBS for 5 min each, and incubated with horseradish peroxidase (HRP)-conjugated goat anti-rabbit IgG (1:5,000 dilution; Sigma, San Francisco, USA) for 1.5 h at room temperature. Finally, the membranes were washed three times with 0.01 M PBS for 5 min. The membrane was exposed to HRP-diaminobenzidine (DAB) substrate (Tiangen, Beijing, China) following the manufacturer’s instructions.

### Immunolocalisation

Polyclonal antisera against r*TpFABP* protein were raised in two 9-week old female parasite-free New Zealand White rabbits (obtained from Laboratory Animal Centre of Sichuan Agricultural University, China) by consecutive subcutaneous inoculation of the r*TpFABP* protein as described by Hu et al. (2002) [12]. The immune sera were obtained by centrifugation at 4,000× *g* for 10 min in room temperature. IgG fractions were isolated using a Protein G affinity chromatography column (Bio-Rad, California, USA) and stored at −80 °C.

The collection of fresh mature metacestodes and adult of *T. pisiformis* was previously described [30]. The parasites were freshly fixed in Bouin’s solution for 24 h and then embedded in paraffin. The sections were cut serially at 7 μm thickness using a slicer (Leica, Wetzlar, Germany). Immunolocalisation was carried out using a streptavidin biotin complex-peroxidase (SABC-POD) with rabbit IgG kit (Boster, Wuhan, China) according to the manufacturer’s protocol. The purified IgG fractions against *TpFABP* were diluted to 200 times. Peroxidase activity was visualised by incubating sections with a DAB-Plus Kit (Boster, Wuhan, China). Finally, the slides were counterstained with Mayer’s haematoxylin, examined with an optical microscope and photographed (Nikon, E800).

### Collection of sera

Three healthy rabbits were used as the negative control. Positive polyclonal antisera against r*TpFABP* protein were as described above.

Experimental sera were collected from seven 90-day old healthy white female New Zealand White rabbits (*Oryctolagus cuniculus*) sourced from the Laboratory Animal Centre of Sichuan Agricultural University, China. The rabbits were orally infected with 5,000 mature viable *T. pisiformis* eggs. Serum samples were collected every 7 days. The rabbits were humanely sacrificed (50 mg/kg ketamine and 100 mg/kg sodium pentobarbital [Sigma, San Francisco, USA]) at 49 days post-infection. Necropsies were as performed as previously described [3].

Test serum samples (*n* = 169) were collected from rabbits from a local slaughterhouse, and the serum was separated and stored at −20 °C. The abdominal cavity of rabbits was examined for the presence of *T. pisiformis* cysticerci, as previously described.

Sera from rabbits (*n* = 22, provided by the Department of Parasitology, Veterinary College, Sichuan Agricultural University, China) infected with *Sarcoptes scabiei* (seven cases), *Psoroptes cuniculi* (eight cases), *Eimeria* spp. (four cases) and *Passalurus ambiguus* (three cases) were used to test cross-reactivity.

### Dot-ELISA

Total *T. pisiformis*-specific IgG antibodies of rabbits were detected by dot-ELISA using r*TpFABP* as the test antigen following the methodology described by Piña et al. (2011) [21] with some modifications. Briefly, 50, 100 and 200 ng of the purified antigen (10 μg/mL) were dotted on marked circular regions at the centre of each NC strip. The positive polyclonal sera (1:50, 1:100, 1:200) and goat anti-rabbit IgG-HRP conjugate (1:7000) (Sigma, San Francisco, USA) were diluted using PBS Tween-20 and 5% (w/v) non-fat milk. ELISA dots were detected by HRP-DAB (Invitrogen, Shanghai, China). The visual reading by two independent observers was the same for all tests, and no difference in colour intensity was observed. In addition to the background colour, a tan-yellow reaction was designated as a positive result. The best dilution of r*TpFABP* antigen and rabbit sera was determined by the colour reaction intensity in the positive dot-ELISA.

The remainder of the experimental and test sera were detected by dot-ELISA and described as above. The percentage sensitivity was calculated as dot-ELISA positive × 100/true positive, and the percentage specificity was calculated as dot-ELISA negative × 100/true negative [28].

## Results

### Sequence analysis

**Figure 1.**
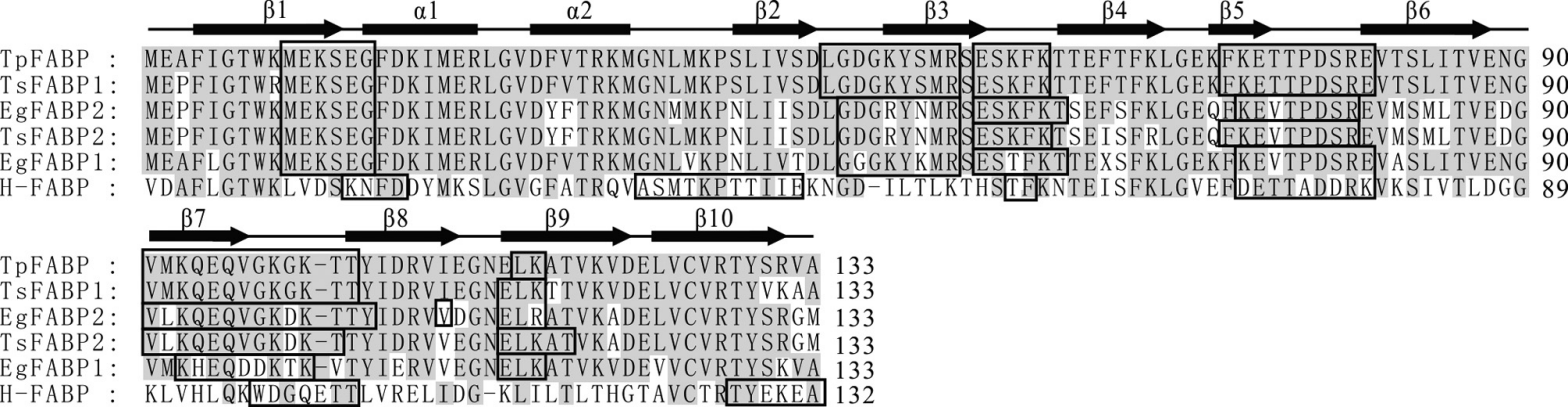
Structural analysis of *TpFABP*. Alignment of the amino acid residue sequences of *Taenia pisiformis FABP* with *T. solium* and *Echinococcus granulosus* in primary structures. The secondary structure of the *TpFABP* amino acid residue sequence was predicted and is shown at the top of the alignment. The light-grey shading indicates the identical amino acid sequences, and locations of linear B-cell epitopes are marked with open boxes. *TpFABP*1, GU205472; *TsFABP*1, HQ259679; *TsFABP*2, AFS64570; *EgFABP*1, 1O8V_A; *EgFABP*2, AAK12095; *H-FABP*, NP_004093.

The *TpFABP* cDNA sequence consisted of an open-reading frame of 402 bp encoding a putative protein with 133 amino acid residues. The results of initial BLASTx searches with *TpFABP* at National Centre of Biotechnology Information (NCBI) showed that the amino acid sequence of *TpFABP* shared 95% identities with *TsFABP*1, 85% with *Echinococcus granulosus FABP*1 (*EgFABP*1), 84% with *TsFABP*2 and *EgFABP*2, and 39.8% with heart-*FABP* (H*-FABP*). In addition, the available FABP sequences from *Oryctolagus cuniculus*, including FABP1 (XM_002709637), FABP2 (intestinal-like, XM_002717226), FABP3 (XM_002716060), FABP7 (brain, XM_002714798), FABP9 (testis, XM_002710656) and FABP12-like (XM_002710657) shared 24–41% identities with the amino acid sequence of *TpFABP*. The protein secondary structure demonstrated a characteristic composition: two antiparallel *α*-helices (including 13 residues) and 10 *β*-strands (including 67 residues; Figure 1). There were six locations of linear B-cell epitopes, including MEKSEG (residues 10–15), LGDGKYSMR (residues 45–53), ESKFK (residues 55–59), KFKETTPDSRE (residues 70–80), VMKQEQVGKGKTT (residues 91–103) and LK (residues 114–115).

### r*TpFABP* expression and western blotting

**Figure 2.**
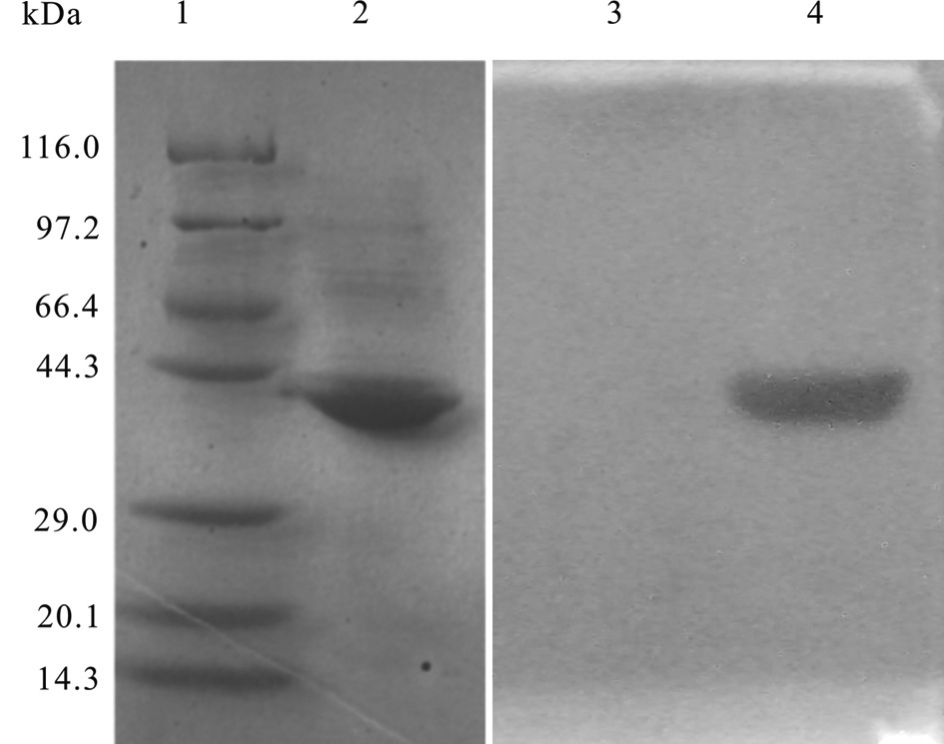
Expression of r*TpFABP* and identification by rabbit antisera in western blotting. Lane (1) molecular weight markers; (2) purified r*TpFABP* protein; (3) r*TpFABP* protein reacted with negative rabbit serum (1:100 v/v dilutions) by western blotting analysis; (4) r*TpFABP* protein reacted with rabbit antisera (1:100 v/v dilutions) by western blotting analysis. Molecular masses (kDa) are indicated on the left.

r*TpFABP* was successfully expressed in *E. coli* strain BL21 (*DE3*). The molecular weight of the recombinant protein was about 36 kDa, and the solubility of r*TpFABP* protein was identified as inclusion bodies. r*TpFABP* was recognised by rabbit *T. pisiformis* cysticercosis antisera in western blotting analysis (Figure 2).

### Immunolocalisation of *TpFABP*

**Figure 3.**
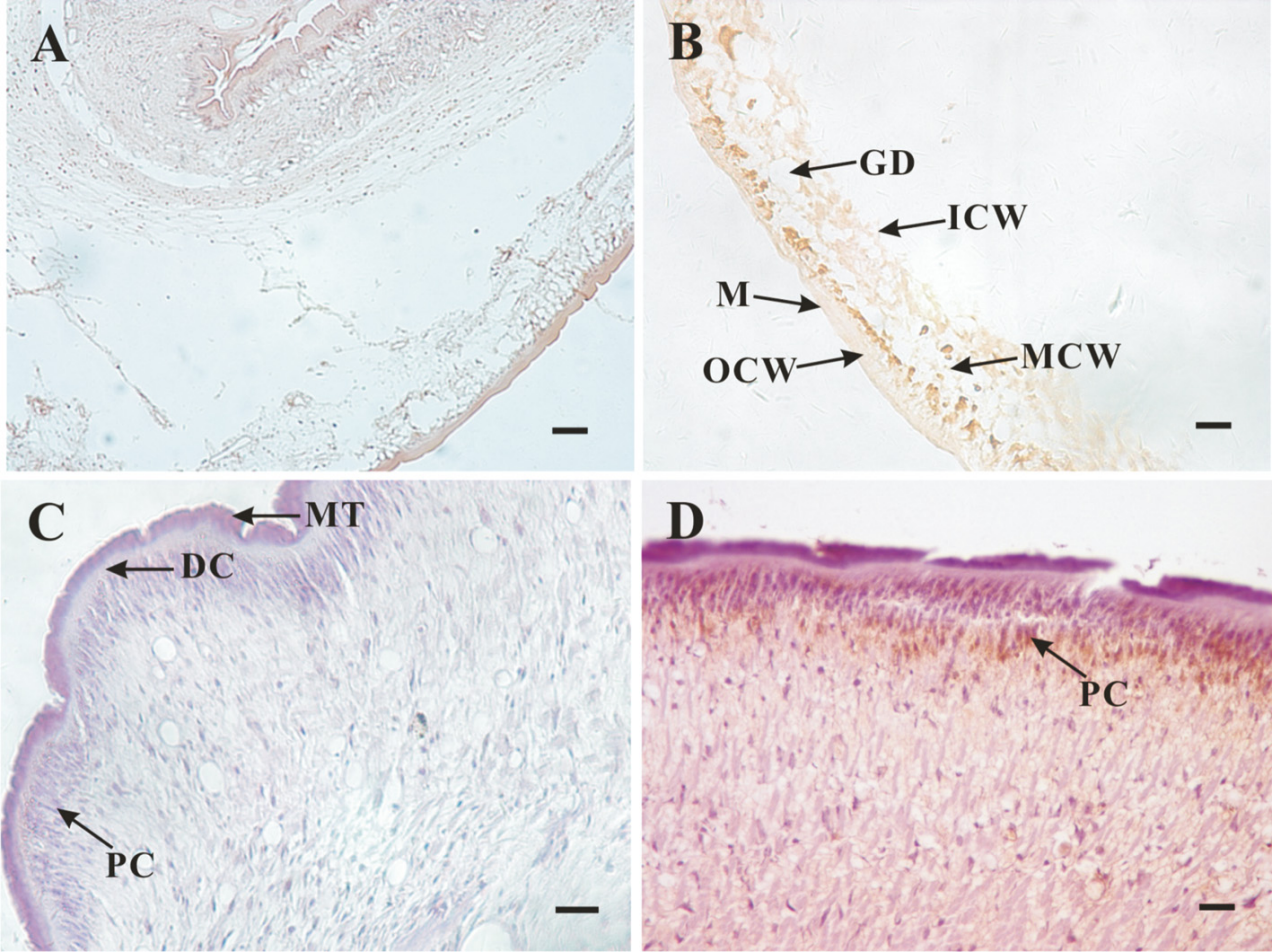
Immunolocalisation of *TpFABP* in *T. pisiformis* tapeworm and cysticercus. The yellowish-brown tint shows the *TpFABP* protein location. (A) negative sera in cysticercus; (B) antisera in cysticercus; (C) negative sera in adult tapeworm; (D) antisera in adult tapeworm. Arrows indicate the areas of the parasite: MT, microthrix; DC, distal cytoplasm; PC, perinuclear cytoplasm; GD, gathering duct; M, microtrichia; ICW, inside the layer of cystic wall; OCW, outer layer of cystic wall; MCW, middle layer of cystic wall. Scale bars: 20 μm.

*TpFABP* was localised in perinuclear cytoplasm (PC) of adult *T. pisiformis* proglottids (Figure 3). Furthermore, the positive signal was observed in the parenchyma of the bladder wall of the cysticercus, and intensely localised in outer layer of cystic wall (OCW) and middle layer of cystic wall (MCW).

### Dot-ELISA

**Figure 4.**
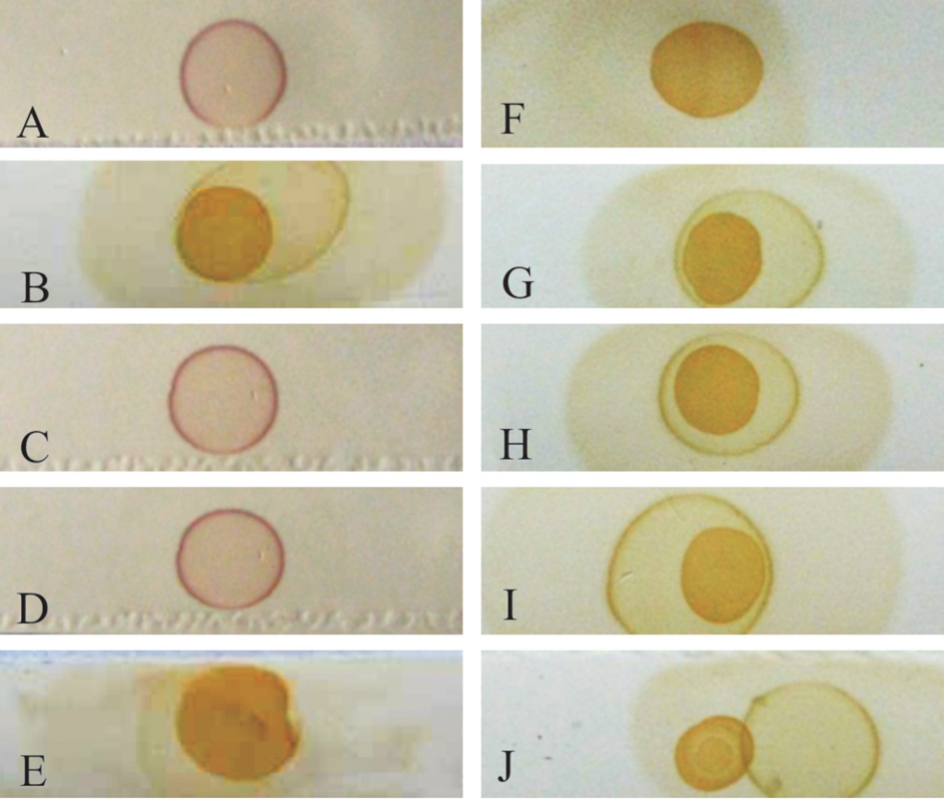
Dot-ELISA of naturally infected rabbit experimental sera with r*TpFABP*. The tan-yellow tint shows the positive reaction: A, negative control sera; B, positive antisera; C, sera at 0 day post-infection; D, sera at 7 days post-infection; E, sera at 14 days post-infection; F, sera at 21 days post-infection; G, sera at 28 days post-infection; H, sera at 35 days post-infection; I, sera at 42 days post-infection; J, sera at 49 days post-infection.

Combinations of various amount of antigens tested with various dilutions of positive polyclonal sera did not show any differences (data not shown). Therefore, 200 ng of r*TpFABP* antigen and 1:100 of sera in each strip were determined to be the optimal combination for the full set of sample tests. The experimental sera were found to be positive at 14 days post-infection, and remained through to 49 days post-infection (Figure 4) when the rabbits were sacrificed.

Of the 169 rabbits sampled from the slaughterhouse, *T. pisiformis* cysticerci were found in 55 rabbits by necropsy, but it was not present in 114 rabbits. Fifty-four rabbits tested positive and 105 were negative by dot-ELISA. Thus, the sensitivity and specificity of dot-ELISA using r*TpFABP* antigen to detect *T. pisiformis* cysticercus were 98.2% (54/55) and 92.1% (105/114), respectively.

There was no cross-reaction between r*TpFABP* and the positive sera of *Sarcoptes scabiei*, *Psoroptes cuniculi*, *Eimeria* spp. and *Passalurus ambiguus*.

## Discussion

Nine groups of *FABP*s have been identified in mammals with variable primary structures (identity, 20–70%), but all the members of this family share a superimposable tertiary structure [1]. In our study, *TpFABP* amino acid sequence shared the highest identity (95%) with *TsFABP*1 in primary structure, and had the 10-stranded *β*-barrel fold, typical for the family of intracellular lipid-binding proteins [15]. Six locations of linear B-cell epitopes between *TpFABP*, *TsFABP*1, *TsFABP*2, *EgFABP*1 and *EgFABP*2 had a similar distribution. Together, these suggest a common conservation of this family of genes within cestode parasites as well as a possible common ancestral gene. However, low identities (23.88–41.04%) between the available FABP amino acid sequences from rabbit and the *TpFABP* from *T. pisiformis* indicated that they would not share a common ancestral gene.

The adult stages of parasitic platyhelminths are dependent on carbohydrates for their energy metabolism [26], but a functional *β*-oxidation pathway has not been demonstrated in cestodes [24]. FABPs synthesise most of their own lipids de novo by combining hydrophobic groups to help platyhelminths, especially long-chain fatty acids and cholesterol [19]. Meanwhile, FABPs have been described as intracellular carriers of fatty acid (FA) [7]. *EgFABP*1 is specifically expressed in the protoscolex larval stage and associated with protoscolex larval development [8]. Abundant expressions of *TsFABP*1 and *TsFABP*2 were found in the canal region of adult *T. solium* [16], and *TsFABP*1 also was positive in subtegumental cytons of tissue sections from cysticerci from *T. pisiformis* [14]. *TsFABP*1 has been demonstrated to be involved in the transport of several fatty acids required for *T. solium* nourishment. It is plausible that *TsFABP*1 is involved in the mechanism by which FAs are mobilised from the translocation site on the tegument membrane, to other cellular compartments in the syncytium. In this study, the positive distribution of *TpFABP* was similar to *T. crassiceps FABP* in cysticerci [14], and was widely distributed in the parenchyma of the bladder wall in the cysticerci. The localisation of *TpFABP* in the cysticerci suggested that the cystic wall layer might be a primary location in the scolex where FA uptake occurs. In tissue sections of adult *T. pisiformis*, the positive staining distribution was the same as *T. solium FABP*1 [14]. *TpFABP* is probably involved in the uptake and transport of fatty acid molecules in the perinuclear cytoplasm to maintain the survival of adult *T. pisiformis*. Thus, the biological role of *TpFABP* in *T. pisiformis* may be similar to that of *FABP*1 in *T. solium* [9]. With guaranteed supply of fatty acids for survival by abundant fatty acid-binding proteins, parasites can adjust their biological mechanism to adapt to a changing environment.

Because *T. pisiformis* infection in rabbits is not associated with specific clinical symptoms, it is difficult to detect it in the early infective stage (up to 7 weeks post-infection). This stage involved adherence of the oncosphere to and migration across the intestinal wall, followed by transport to the liver parenchyma via the circulatory system. The oncosphere finally migrates to the abdominal cavity of rabbits [18]. Detection of the antibody against *T. pisiformis* cysticerci could be useful for early detection and treatment of this infection. Circular antibodies in experimental *T. pisiformis* infections of rabbits have been previously investigated [6], which indicated that antibodies were detected in rabbit sera by 2 weeks post-infection using the in vitro-derived *T. pisiformis* metacestode antigen. In addition, Wang et al. (2009) [20] investigated the dynamic profile of antibodies in rabbits experimentally infected with *T. pisiformis* using crude antigen from mature metacestodes, and found that the antibody levels started to increase at week three post-infection and were up to the highest level at week eight post-infection. In our study, dot-ELISA of r*TpFABP* was successfully established to detect rabbit *T. pisiformis* cysticercosis under the optimum conditions. The antibodies in experimental sera could be detected by dot-ELISA in the early stage of infection (14 days), and lasted 7 weeks (49 days) post-infection. The dot-ELISA was developed in this study with a high sensitivity (98.2%) and specificity (92.1%) for 169 tested sera samples when compared with the results of necropsy. The cross-reactivity of several parasites of rabbit, a potential probability of false positives, was also carried out, and no cross-reactivity was observed with our panel of positive sera of other parasites. Thus, r*TpFABP* antigen can detect specifically *T. pisiformis* cysticercosis in tested rabbits.

Together, the data shows that r*TpFABP* is a suitable diagnostic antigen, and the results of study demonstrate the efficacy of the FABP-based dot-ELISA for potential detection of *T. pisiformis* cysticercosis in rabbit.
